# UVB irradiation does not directly induce detectable changes of DNA methylation in human keratinocytes

**DOI:** 10.12688/f1000research.2-45.v1

**Published:** 2013-02-13

**Authors:** Christoph Lahtz, Sang-In Kim, Steven E Bates, Arthur X Li, Xiwei Wu, Gerd P Pfeifer

**Affiliations:** 1 Department of Cancer Biology, Beckman Research Institute, City of Hope, Duarte, 91010, USA; 2 Department of Information Sciences, Beckman Research Institute, City of Hope, Duarte, 91010, USA; 3 Department of Molecular Medicine, Beckman Research Institute, City of Hope, Duarte, 91010, USA

## Abstract

Unprotected exposure to UVB radiation from the sun and the resulting DNA damage are thought to be responsible for physiological changes in the skin and for a variety of skin cancers, including basal cell and squamous cell carcinoma and malignant melanoma. Although the mutagenic effects of UVB have been well documented and studied mechanistically, there is only limited information as to whether UV light may also be responsible for inducing epigenetic changes in the genome of exposed cells. DNA methylation is a stable epigenetic modification involved in gene control. To study the effects of UVB radiation on DNA methylation, we repeatedly exposed normal human keratinocytes to a UVB light source. After a recovery period, we analyzed global DNA methylation patterns in the irradiated and control cells using the methylated-CpG island recovery assay (MIRA) method in combination with high-resolution microarrays. Bioinformatics analysis revealed only a limited number of possible differences between UVB-exposed and control cells. However, these minor apparent changes could not be independently confirmed by bisulfite sequencing-based approaches. This study reveals that UVB irradiation of keratinocytes has no recognizable global effect on DNA methylation patterns and suggests that changes in DNA methylation, as observed in skin cancers, are not immediate consequences of human exposure to solar UVB irradiation.

## Introduction

Solar UV light is divided into three wavelength categories: UVA with a wavelength between 320 nm and 400 nm, UVB with a wavelength between 280 nm and 320 nm, and far UV light (UVC) with a wavelength between 100 nm and 280 nm. UVC radiation is filtered by the atmosphere and technically does not exist on the earth’s surface. However, a fraction of UVB and much of the UVA wavelength radiation reach the surface of the earth and have been implicated in skin cancers and other acute and chronic aberrations of the skin such as sunburn and premature skin aging, respectively. Most of the skin cancer-causing effects of sunlight have been ascribed to UVB radiation with a smaller contribution from UVA ^1, 2^. UVB induces direct DNA damage through the formation of cyclobutane pyrimidine dimers (CPDs) and another dipyrimidine lesion, the (6-4) photoproduct ^3– 6^. Of these two types of lesions, the CPD is thought to be responsible for the majority of mutations induced by UVB or sunlight irradiation ^7, 8^. These mutations are characterized by a preponderance of C to T transition mutations at dipyrimidine sites containing cytosine, for example 5′TC and 5′CC. Very often, 5-methylcytosines (mC), when part of a dipyrimidine sequence, are seen as preferential sites of CPD formation and also as preferential mutational target sites in mammalian cells ^9– 11^. These types of mutations, i.e. C or mC to T mutations at 5′TC, 5′CC, 5′TmC, and 5′CmC, are recognized as the major mutational events in human skin cancers, both in specific genes ^4^ and in large-scale genomic sequencing studies analyzing thousands of different genes simultaneously ^12– 14^.

Besides mutations, the other frequent change observed at the DNA level of skin cancer genomes is the aberration of DNA cytosine methylation patterns. Like most cancer types, both nonmelanoma and melanoma skin cancers are characterized by substantially aberrant DNA methylation ^15– 21^. DNA hypermethylation is widespread and affects many CpG islands, which are defined as CpG dinucleotide-rich genomic sequences, often found around promoters of genes. This DNA hypermethylation can affect hundreds of genes in individual tumors, sometimes producing a cancer-driving event, for example if genes involved in growth control and/or DNA repair are involved ^22, 23^. Although the methylation changes were first described many years ago, the mechanisms of cancer-associated DNA hypermethylation or hypomethylation have remained obscure. One model proposes that environmental influences, in the form of exposure of humans to either chemicals or radiation, may produce these aberrant DNA methylation events ^24^. For example, one could conceive a scenario in which this exposure induces a signaling cascade and transcriptional changes inside cells that would affect DNA methylation patterns, for example by modulating the DNA methylation machinery or the chromatin state at genes that become susceptible to methylation. Such UV-induced heritable DNA methylation changes could lead to an altered phenotype and could provide a selective advantage to cells, perhaps when combined with UVB-induced mutations, and could thus be viewed as a tumor-driving event. In this study, we examined this hypothesis by exposing human keratinocytes chronically to UVB radiation and by assessing DNA methylation patterns on a genomic scale following UV exposure of cells and a recovery period.

## Materials and methods

### Cells and biological materials

Normal human keratinocytes (Clonetics; San Diego) were grown in EpiLife Medium (Invitrogen; Carlsbad, CA). The restriction enzyme used for combined bisulfite restriction analysis (COBRA), Taq ^α^I (5′-TCGA-3′), was obtained from New England Biolabs (Ipswich, MA).

### UVB radiation treatment

The UVB source we used consisted of three fluorescent light tubes (Philips TL 20 W/12R) filtered through a cellulose acetate sheet, which eliminates wavelengths below 295 nm. The source has a peak spectral emission at 312 nm. The keratinocytes were grown in 150 mm cell culture dishes. The cells were irradiated with doses of 260 J/m ^2^ (high dose) and 130 J/m ^2^ (low dose) of UVB after the medium had been removed and cells had been washed three times with phosphate buffered saline. After UVB exposure, the cells were grown in new culture medium for three days. They were then irradiated again, and then nine more times with a two or three day recovery time between each irradiation cycle. After the final irradiation dose was delivered, the cells were grown for a recovery period of eight days or 18 days.

### DNA isolation

The cells were trypsinized and collected by centrifugation. After a proteinase K treatment, DNA was isolated with a standard phenol/chloroform extraction method followed by ethanol precipitation ^25^.

### MIRA and microarray

To detect potential genome-wide changes in DNA methylation patterns after the UVB treatments, the methylated-CpG island recovery assay (MIRA) combined with microarray analysis was used as described previously ^26, 27^. Nimblegen’s Signalmap program was used to visualize the DNA methylation data and for generation of profiling snapshots.

### Bioinformatics analysis

Loess normalization was applied to the raw intensity files of each array to correct intensity-dependent dye bias and obtain log2 ratios between MIRA and input samples. Then the log2 ratios across all the samples were quantile-normalized. Probes were considered positive if their normalized log2 ratios were above 2-fold. Peaks in each sample were called if a minimum of four consecutive positive probes were present with either one gap or no gaps. To identify hypermethylated and hypomethylated targets in UVB-treated samples vs. untreated control samples, the average probe log2 ratio signals within the peaks identified in each UVB-treated sample were compared to the untreated samples. Only the peaks with an average log2 ratio signal difference of more than log2(3) were considered hypermethylated or hypomethylated peaks. These differential peaks were annotated to the Refseq transcript database downloaded from the UCSC genome database. The microarray data have been deposited into the GEO database (accession number GSE42943).

### DNA methylation analysis by COBRA

Candidate loci were investigated by combined bisulfite restriction analysis (COBRA) ^28^. PCR was performed with primers and conditions listed in Supplementary Table S1 and Supplementary Table S2. Briefly, COBRA-PCR was performed with bisulfite-converted DNA-specific primers using 50 ng of bisulfite- modified genomic DNA as template for 60 cycles after a 15 min incubation at 95°C, then 30 s at the T _A_ (see Table S2) and 30 s at 72°C in a 25 µl volume containing 5 nmol dNTPs, 20 pmol primers, and 1.25 units of Hot start *Taq* DNA polymerase (Qiagen, Valencia, CA). Five microliters of the PCR product was analyzed on a 2% agarose gel. Equal amounts of PCR product were digested with the restriction enzyme Taq ^α^I (5′-TCGA-3′).

### Bisulfite sequencing

COBRA PCR products were created as described above. The PCR products were ligated into a cloning vector (TOPO ^®^ Cloning Kit; Invitrogen, Grand Island, NY, or the pGEM ^®^-T-Easy Kit, Promega, Madison, WI) and transformed into competent cells. Different clones were picked randomly and the plasmids were isolated and sequenced. For the analysis of methylated and unmethylated cytosines, the free software program Bioedit was used.

## Results

### Genome-scale DNA methylation analysis of UVB-irradiated keratinocytes

We irradiated human keratinocytes with two different doses of UVB, 130 J/m ^2^ and 260 J/m ^2^. These doses were well tolerated by the cells and did not produce overt losses in cell viability. Cells were irradiated chronically (11 times total) with these doses with a 2–3-day recovery period between each irradiation cycle. Controls included cells that were not irradiated and cells irradiated once with 130 or 260 J/m ^2^ of UVB, but then harvested immediately after the irradiation.

**Figure 1.**
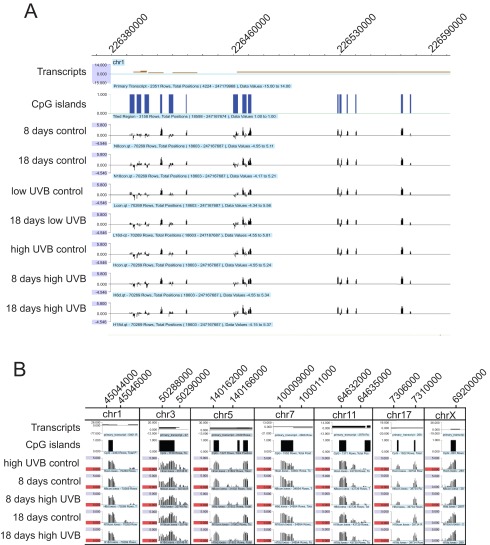
DNA methylation peaks in control and UVB-irradiated keratinocytes. **A**. A random segment of chromosome 1 is shown to indicate the reliability of the method and to show the uniform peaks between control and UVB-irradiated samples. The positions of transcripts and CpG islands are indicated. The samples labeled ‘low UVB control’ and ‘high UVB control’ represent DNA immediately harvested following a one-time irradiation of cells with either 130 J/m ^2^ (low) or 260 J/m ^2^ (high) of UVB. Samples labeled ‘8 days control’ or ’18 days control’ were grown for the same time periods as the irradiated cells but were never irradiated. Samples labeled ‘18 days low UVB’ or ‘18 days high UVB’ were chronically irradiated with 130 J/m ^2^ (low) or 260 J/m ^2^ (high) of UVB followed by an 18 day recovery period. The sample labeled ‘8 days high UVB’ was chronically irradiated with 260 J/m ^2^ of UVB followed by an 8 day recovery period. **B**. Methylation peaks are shown for randomly selected genomic regions on chromosomes 1, 3, 5, 7, 11, 17 and X. The positions of transcripts and CpG islands are indicated. The chromosomal coordinates are shown above each snapshot.

**Figure 2.**
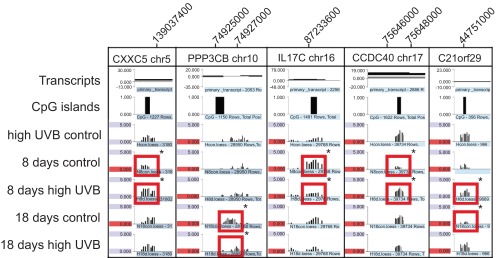
Apparent DNA methylation differences in control and UVB-irradiated keratinocytes. Methylation peaks are shown for the genes *CXXC5*, *PPP3CB*, *IL17C*, *CCDC40*, and *C21orf29*. The positions of transcripts and CpG islands are indicated. The chromosomal coordinates are shown above each snapshot. The signals framed by the red rectangles are significantly (*) differentially methylated pairs of a control sample and a UVB-treated sample, as determined by bioinformatics analysis. The description of the samples is shown in Figure 1.

After the irradiation and final recovery times of eight or eighteen days, DNA was isolated from the cells. DNA was sonicated and the methylated fraction of the genome was enriched by use of the methylated CpG island recovery assay (MIRA) technique ^29^. The methylated fraction was hybridized relative to input DNA onto NimbleGen CpG island plus promoter arrays. These arrays cover all ~28,000 CpG islands of the human genome and all Refseq gene promoters from -2.4 kb to +0.6 kb relative to the transcription start site. Analysis of methylation patterns using NimbleGen’s Signalmap display software indicated the excellent reproducibility of the data ( Figure 1). The patterns of methylation peaks were remarkably similar between all four controls and all three UVB-irradiated samples. Bioinformatics analysis was used to identify potential differences between the control and treatment groups. Methylation peaks were identified as described in Materials and Methods. Peaks with an average log2 ratio signal difference of more than log2(3) were considered as hypermethylated or hypomethylated peaks, respectively. Table 1 summarizes the number of differences identified between UVB and control treatment groups. Most comparisons revealed only a handful of differences and the numbers of differential peaks were generally below 100 for each comparison. In fact, comparison between two controls, non-irradiated cells grown for 18 days or 8 days (N18con vs. N8con), respectively, showed a greater number of differences than any comparison between a UVB-irradiated and a control sample ( Table 1). Nonetheless, a small number of differential peaks could clearly be detected on SignalMap profiles ( Figure 2). We show examples for the genes *CXXC5*, *PPP3CB*, *IL17C*, *CCDC40* and *C21orf29*, where either hypermethylation or hypomethylation in a UVB-irradiated sample was observed ( Figure 2).

**Table 1.** Hypermethylated and hypomethylated candidate gene targets in human keratinocytes following UVB irradiation.

Comparison	Hyper ^b^	Hypo ^b^
H8d vs. Hcon ^a^	2	5
H8d vs. N8con	14	81
H18d vs. Hcon	2	4
H18d vs. N18con	7	51
L18 vs. Lcon	5	29
L18d vs. N18con	7	45
Hcon vs. N18con	16	50
Hcon vs. N8con	25	62
N18con vs. N8con	74	106

^a^Treatments:

Lcon: 130 J/m ^2^ UVB once, cells harvested immediately after irradiation

Hcon: 260 J/m ^2^ UVB once, cells harvested immediately after irradiation

N8con: no UVB, cells harvested after 8 days

N18con: no UVB, cells harvested after 18 days

H8d: 260 J/m ^2^ UVB, 11 times, cells harvested 8 days following final dose

L18d: 130 J/m ^2^ UVB, 11 times, cells harvested 18 days following final dose

H18d: 260 J/m ^2^ UVB, 11 times, cells harvested 18 days following final dose

^b^Hyper- and hypomethylated peaks were defined as described in Materials and Methods

### Bisulfite-based DNA methylation assays

**Figure 3.**
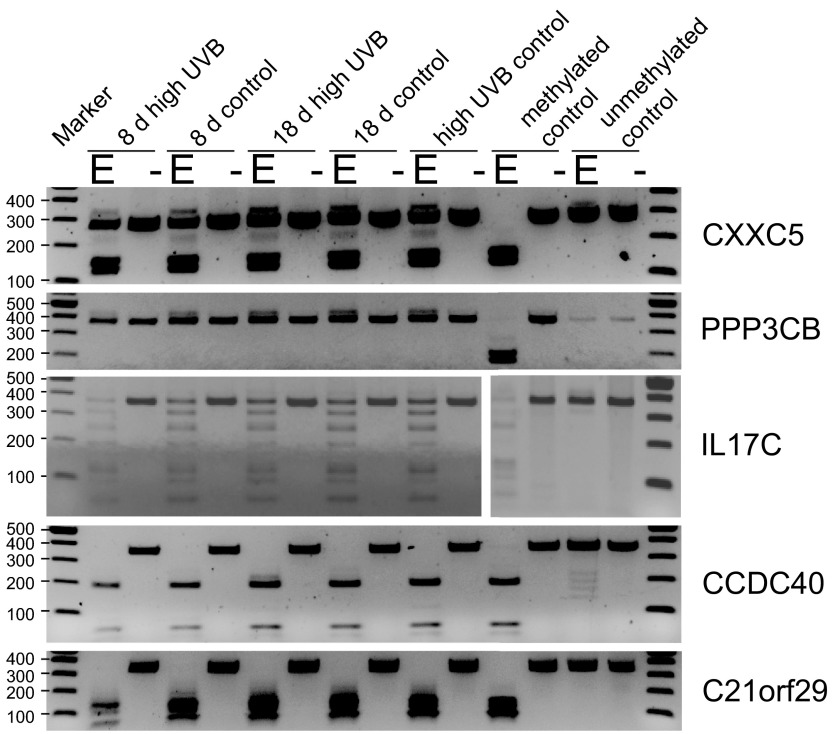
DNA methylation analysis by COBRA of candidate differentially methylated genes. COBRA analysis of restriction enzyme sites indicates no substantial difference in DNA methylation in UVB-irradiated cells versus controls. Samples labeled ‘8 d high UVB’ and ‘18 d high UVB’ were chronically irradiated with 260 J/m ^2^ of UVB followed by an 8 day or 18 day recovery period, respectively. Samples labeled ‘8 d control’ or ‘18 d control’ were grown for the same time periods as the irradiated cells but were never irradiated. The samples labeled ‘high UVB control’ represent DNA immediately harvested following a one-time irradiation of cells with 260 J/m ^2^ of UVB. The genes *CXXC5*, *PPP3CB*, *IL17C*, *CCDC40* and *C21orf29* were analyzed by Taq ^α^I digestion. The PCR products were cleaved with the enzyme (E) or were left uncleaved (-). The methylated and unmethylated control samples at the right side of the gel panels were fully CpG-methylated and unmethylated control DNAs.

To verify the apparent methylation differences observed by microarray analysis, we performed sodium bisulfite conversion-based DNA methylation assays. COBRA analysis is shown for the genes *CXXC5*, *PPP3CB*, *IL17C*, *CCDC40* and *C21orf29* in Figure 3. Cleaved molecules in these assays indicate methylated restriction sites that are resistant to bisulfite conversion and remain cleavable by the CpG-targeting restriction enzyme after PCR. Uncut molecules represent unmethylated DNA fragments. The COBRA assays indicated that the methylation patterns were the same or very similar between DNA isolated from control cells and DNA from UVB-irradiated cells.

**Figure 4.**
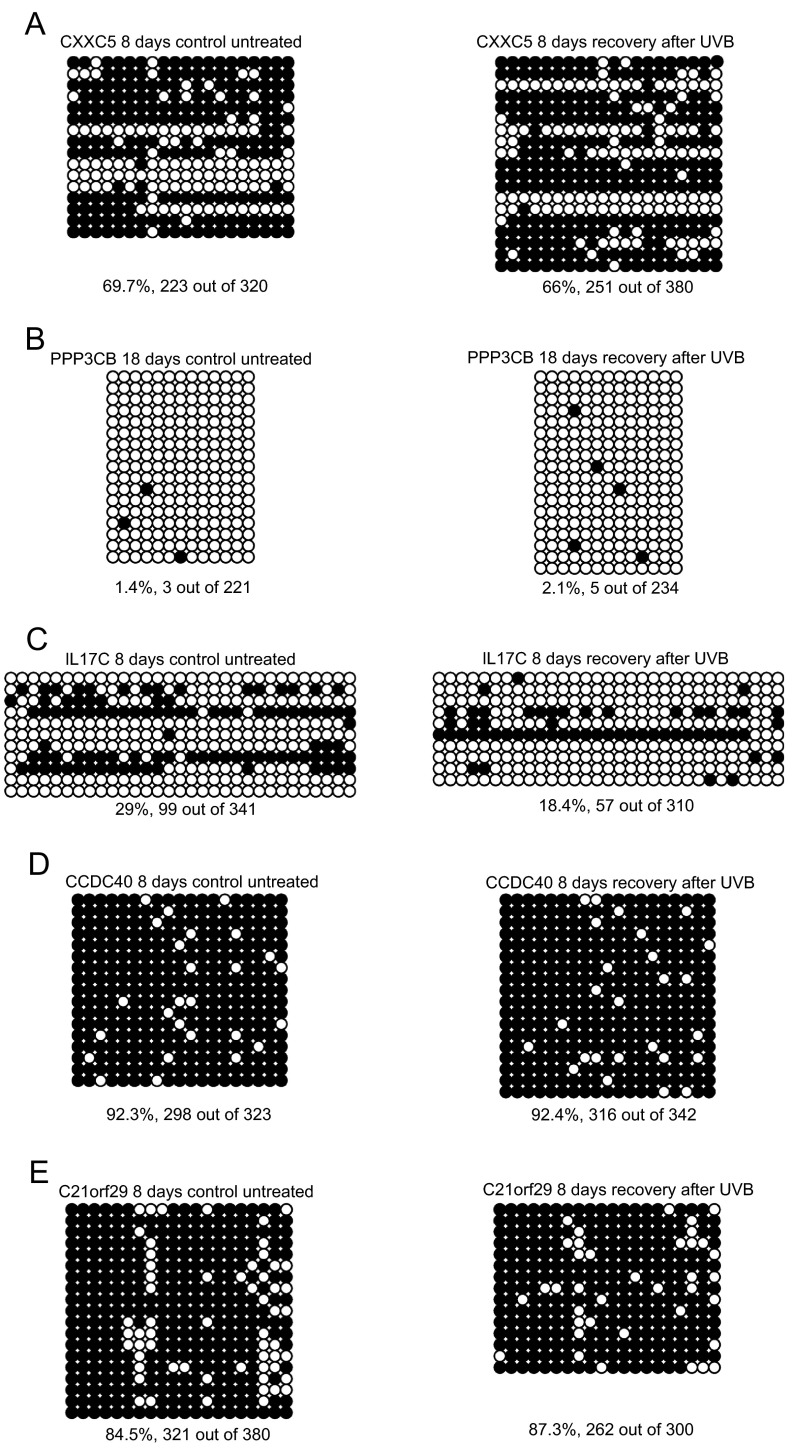
DNA methylation analysis by bisulfite sequencing of candidate differentially methylated genes. Bisulfite sequencing data are shown for the genes *CXXC5* ( **A**), *PPP3CB* ( **B**), *IL17C* ( **C**), *CCDC40* ( **D**), and *C21orf29* ( **E**). The chronic UVB dose was 260 J/m ^2^. Open circles represent unmethylated CpG sites and black circles indicate methylated CpG sites. The percentage of methylated sites is indicated below the sequence data for individual cloned molecules.

COBRA assays, although generally indicative of the methylation status of a genomic target, can score only a limited number of CpG sites. Therefore, we performed sodium bisulfite sequencing to provide the methylation status of all CpG within the amplified target fragments. These assays also indicated no substantial difference between control and UV-irradiated cells ( Figure 4). Similar methylation patterns were observed for the five different gene targets, regardless of whether the cells were UVB-irradiated or not. A somewhat lower frequency of methylated CpG sites was observed for the *IL17C* gene in UVB-irradiated cells (18.4% versus 29%). However, these results may be biased by the few molecules in the population that had almost every CpG methylated. Taken together, our results suggest that the rather small number of differential peaks observed by bioinformatics analysis were false positives. Such small numbers of false positive differences can be expected when comparisons are made for over 28,000 CpG islands and about 20,000 Refseq promoters.

Bisulfite sequencing data Raw sequence reads for bisulfite sequencing data shown in Figure 4

## Discussion

The field of environmental epigenetics has recently received much attention ^24^. Environmental genotoxic or non-genotoxic carcinogens, theoretically at least, could alter the epigenome at the levels of DNA methylation or histone posttranslational modifications. Such carcinogen-induced epigenetic effects may be heritable and may contribute to the etiology of human cancer and other diseases. Effects of environmental exposure on the epigenome may be direct or indirect. For example, indirect effects could be produced by carcinogen-induced gene mutations in a gene target encoding an epigenetic modifier protein thus leading to an epigenetic defect. Mutation of histone modifying enzymes or DNA modification enzymes would fall into this category. Such mutations are indeed observed in several types of human cancer ^30^ including melanoma ^13^. A more immediately acting but still indirect effect of an environmental exposure on epigenetic marks may occur via signaling cascades, e.g. through a DNA damage response pathway, impinging on the expression levels of epigenetic modifiers or on local chromatin structure at a susceptible gene locus, thereby modulating DNA methylation patterns.

On the other hand, exposure of target cells to an environmental agent may produce a more direct effect on the epigenome if the effect is mediated by DNA damage. In the case of UV irradiation, an interesting photochemical deamination and demethylation pathway of 5-methylcytosine has been described ^31^ but its biological relevance has remained unknown. Furthermore, it has been shown several decades ago that UVB irradiation can inhibit DNA methyltransferases *in vitro* ^32^. UVB-induced pyrimidine dimers may also alter nucleosome association with DNA ^33^, thereby potentially changing DNA methylation patterns at a susceptible gene locus. Repair of the pyrimidine dimer damage may remove methylated cytosines during the excision repair step thus contributing potentially to altered DNA methylation.

However, our data are inconsistent with proposals that UVB can change DNA methylation patterns heritably as a direct consequence of chronic exposure. Rather, our sensitive and specific genome-scale analysis of DNA methylation patterns in UVB-exposed keratinocytes has not uncovered any substantial changes in DNA methylation patterns, either 8 days or 18 days following chronic exposure of the cells to UVB. The few differences observed on the microarray could not be confirmed by bisulfite-based sequence analysis thus suggesting that these differences were false positives. While we cannot exclude the possibility that more drastic UV doses or a longer waiting time may produce some changes, the data presented here make it questionable that the many DNA methylation differences observed in human skin cancers are a direct consequence of exposure of skin cells to UVB radiation from the sun. The numerous skin cancer-associated methylation changes must therefore be events occurring later during the cell transformation process. As discussed earlier, the vast majority of the methylation differences seen in cancer are likely passenger events and do not appear to be selected ^22^. These methylation events could be secondary to specific genetic events, as discussed above, or they could represent random or locus-targeted methylation gains or losses occurring over the timeline of enhanced cell proliferation. Alternatively, DNA methylation differences found in skin tumors could be induced by other processes such as inflammation.

Future studies will determine if methylation changes can be induced directly by UVA, a longer wavelength component of sunlight that also has a strong component of oxidative DNA damage ^4, 34^. UVA, however, produces less severe overall levels of DNA damage than UVB and therefore may be even less likely to induce direct DNA damage-dependent changes of methylation patterns. Other skin cell types, in particular melanocytes, will also need to be analyzed to better understand the role of UV irradiation in melanoma pathogenesis.

## Figures and Tables

**Supplementary Table 1.** Oligonucleotide primers.

Gene	Upper Primer (5’ → 3’)	Lower Primer (5’ → 3’)
	Oligonucleotides for combined bisulfite restriction-analysis (COBRA)	
*CXXC5*	GTT TTT GTA TGG ATA AAG GGG TTT T	CTT CCA AAC CAA AAA AAA CCT AAC
*PPP3CB*	TTT TTT TTA TAG AGG GGT TAA GAT	CTC CAT TTT AAA TAA TCC CTT CTC C
*IL17C*	TTA GGA GGA TAG GGT AGG GTT AGA G	AAA AAA TAC CCT CTA AAA AAC ACA C
*CCDC40*	TTT GTA GAT TGA GAT GGA GAA TTT G	CCT AAA AAA CAA AAT AAA AAA CAA CC
*C21orf29*	TTT GTG ATT ATA TGT TTT GGT TTG G	AAC ATC CCC CTT CTC ATA ACT AAA T

**Supplementary Table 2.** PCR parameters.

Gene	Length in base pairs	Annealing temperature (T _A_)	Restriction enzyme	Formamide concentration
*CXXC5*	286	55°C	TaqαI	2%
*PPP3CB*	377	52°C	TaqαI	2%
*IL17C*	367	52°C	TaqαI	2%
*CCDC40*	358	55°C	TaqαI	2%
*C21orf29*	361	50°C	TaqαI	4%
