# Supplementary material for: UVB irradiation does not directly induce detectable changes of DNA methylation in human keratinocytes
Source: F1000Res. 2013 Feb 13;2:45. [Version 1] doi: 10.12688/f1000research.2-45.v1 (PMC3901454; doi:10.12688/f1000research.2-45.v1)
Supplement: Peer review history

# Peer review: Referee response for version 1

**Published:** 2013-03-18

**Authors:** Shisheng Li

**Affiliations:** 1 Louisiana State University, Baton Rouge, LA, USA

This is a scientifically sound and clearly written article. The major conclusion, that UVB irradiation of keratinocytes has no recognizable global effect on DNA methylation, is supported by carefully designed and well executed experiments. The authors made appropriate speculations about the causes and timing for DNA methylation changes observed in human skin cancers. In view of the nature of somatic cancer development, namely stochastic transformation followed by clonal selection, it is also possible that UVB exposures cause DNA methylation change in only a small fraction of the cells, which cannot be detected with techniques that can only measure collective overall changes but not those in individual cells.

I have read this submission. I believe that I have an appropriate level of expertise to confirm that it is of an acceptable scientific standard.

# Peer review: Referee response for version 1

**Published:** 2013-03-08

**Authors:** Tom Tullius

**Affiliations:** 1 Department of Chemistry, Boston University, Boston, MA, USA

This is an interesting paper, and a good example of how apparently negative results can be important to a field. The authors establish, by three different experiments, that the DNA methylation status of human keratinocytes is not affected by UV irradiation. The results are clear from the data presented in the figures, and the authors are careful to provide bioinformatics analysis to back up visual analysis of the experimental data. This work shows that for the case of UV irradiation, the tantalizing possibility that environmental insult leads to heritable epigenetic changes is not operative. This paper represents a good benchmark for future work that investigates the same hypothesis for other environmental insults.

I have read this submission. I believe that I have an appropriate level of expertise to confirm that it is of an acceptable scientific standard.
